# Excessive elevation of serum phosphate during tumor lysis syndrome: Lessons from a particularly challenging case 

**DOI:** 10.5414/CNCS110086

**Published:** 2021-04-16

**Authors:** Prince K. Amaechi, Fredrik Jenssen, Zipporah Krishnasami, Anand Achanti, Tibor Fülöp

**Affiliations:** 1 Department of Inpatient Medicine, Spartanburg Regional Medical Center,; 2 Spartanburg Nephrology Associates, Spartanburg,; 3 Medicine Service, Ralph H. Johnson VA Medical Center, Charleston, and; 4 Department of Medicine – Division of Nephrology, Medical University of South Carolina, Charleston, SC, USA

**Keywords:** acute kidney injury, continuous renal replacement therapy, dialysis disequilibrium syndrome, hypocalcemia, metabolic acidosis, osmolality

## Abstract

Burkitt’s lymphoma is a common cause of tumor lysis syndrome (TLS) and, in the era of aggressive utilization of prophylactic allopurinol and recombinant uricase enzyme, nephrologists are increasingly witnessing monovalent or divalent cation abnormalities without marked uric acid elevation. An 18-year-old male received his 1^st^ cycle of intensive chemotherapy for Burkitt’s lymphoma and developed TLS as defined by the Cairo Bishop criteria. Lactate dehydrogenase peaked at 9,105 U/L (range: 130 – 250) and was accompanied by acute kidney injury, including serum creatinine 2.2 mg/dL on the 4^th^ day with oliguria, hyperkalemia, extreme hyperphosphatemia (21.4 mg/dL), hypermagnesemia, and hypocalcemia. Renal replacement therapy decision was made based on life-threatening electrolyte disturbances. The competing necessity to effectively control hyperphosphatemia and avoid the complication of dialysis disequilibrium syndrome prompted us to perform an initial intermittent hemodialysis with simultaneous intravenous mannitol administration, followed by continuous hemodialysis to manage the continued production of phosphorus from cell lysis. Osmotic stability during the therapy session was affirmatively demonstrated (322, 319 mOsm/kg, respectively). The patient showed excellent tolerance for these therapies and eventually recovered renal function as demonstrated during follow-up visits.

## Introduction 

Tumor lysis syndrome is common in patients with Burkitt’s lymphoma and may occur after initial therapy of high tumor load of lymphomas or leukemias. Burkitt’s lymphoma is one of the most aggressive of the large B-cell lymphomas, and the patient could present with life-threatening electrolyte disturbances as a result of rapid cell breakdown, with release of intracellular proteins and nucleic acids causing hyperkalemia, hyperphosphatemia, hypocalcemia, hyperuricemia, and AKI. This is especially true after the initiation of chemotherapy [1]. Patients may be diagnosed based on laboratory abnormalities (laboratory TLS) or both laboratory and clinical criteria (clinical TLS) [2]. 

Patients with high international prognostic index have poorer prognosis and are more likely to relapse [3]. The guidelines for the management of TLS published by the British Committee for Standard in Hematology, recommend that patients due to receive chemotherapy for any hematological malignancy should undergo a TLS risk assessment (a grade 1B recommendation) [4]. The duration of treatment should be determined by the clinical response, and dialysis, if indicated, should be continued until the recovery of renal function or the resolution of electrolyte abnormalities [4][Table Table1][Table Table2]. 

## Case presentation 

An 18-year-old male received the 1^st^ cycle of intensive chemotherapy for Burkitt’s lymphoma and developed massive tumor lysis syndrome (TLS), the diagnosis confirmed by laboratory as well as clinical criteria, and developed multiple electrolyte disturbances, in particular a very high and rising serum phosphorus level that was difficult to control and posed an immediate danger to life. Lactate dehydrogenase peaked at 9,105 U/L (reference: 130 – 250) and was accompanied by acute kidney injury (AKI) with a serum creatinine level of 2.2 mg/dL by the 4^th^ day along with oliguria (< 400 mL/day), hyperkalemia (6.2 mEq/L), extreme hyperphosphatemia (21.4 mg/dL), hypermagnesemia (3.1 mg/dL), and hypocalcemia (corrected calcium: 7.0 mg/dL). The decision was made to initiate urgent renal replacement therapy based on life-threatening electrolyte disturbances. We utilized an initial session of conventional intermittent hemodialysis (iHD) to control the marked elevation of phosphate, followed by continuous hemodialysis to address the continued release of phosphorus from cell lysis of hematologic malignancy. The initial iHD was a 4-hour session with a blood flow of 200 mL/min and dialysate flow of 400 mL/min with the electrolyte composition of sodium 150 mEq/L and calcium 2.5 mE/L. Additionally, in our case, we utilized a simultaneous mannitol administration of 12.5 g at 2 and 4 hours after the start of hemodialysis to ensure the stability of serum osmolality and prevent potential for dialysis-related disequilibrium syndrome. The serum phosphate improved to 8.7 mg/dL; thereafter, high-volume slow continuous hemofiltration commenced. The initial hemofiltration rate was 30 mL/kg/hour (2 L/h), however the serum phosphate was difficult to control and even rose temporarily within the next 24 hours (13 and 11 mg/dL, respectively), so the rate of hemofiltration was subsequently escalated to 3.5 L/h. The patient showed excellent tolerance for these therapies and eventually recovered renal function as demonstrated during follow-up visits (serum creatinine 0.7 mg/dL). 

### Pathophysiology 

TLS is the logical sequela of the rapid lysis of cells found in rapidly growing malignancies, which overwhelm the homeostatic capabilities of the organism. Hyperphosphatemia directly decreases proximal tubular phosphate absorption via NPi 1a and 1c cotransporters which, in turn increases the excretion of phosphate. Phosphatonins also decrease phosphate reabsorption by suppressing the luminal expression of sodium-phosphate cotransporters [5]. 

Hyperphosphatemia is known to contribute significantly to the development and maintenance of anion gap (AG) metabolic acidosis. Most clinicians learned the mnemonic GOLD PARRK to include hyperphosphatemia as a cause of high AG acidosis, (G for glycols, O for 5-oxoproline, L for lactic acidosis, D for D-lactic acidosis, P for hyperphosphatemia, A for alcohols and acetyl salicylic acid, R for renal failure and rhabdomyolysis, and K for ketoacidosis) [6, 7]. The cellular toxicity from hyperphosphatemia can lead to the clinical catastrophe of hyperphosphatemic and hypocalcemic coma and has been documented as a cause of rapidly fatal respiratory failure [9]. 

Generally, hyperphosphatemia does not frequently necessitate acute treatment in situations where the renal function is well preserved. There is not much guidance in the literature for the management of hyperphosphatemia in the absence of chronic kidney disease or end-stage renal disease. The treatment of acute phosphate nephropathy usually targets improving the excretion of phosphate from the body, either by volume expansion, administration of phosphate binders, or ultimately renal replacement therapy. High serum uric acid levels, on the other hand, can cause endothelial dysfunction via chemokine mechanisms that involve the formation of oxygen free radicals and the activation of the renin-angiotensin system. Additional mechanisms for renal injury from hyperuricemia in TLS include tubular deposition and subsequent intratubular obstruction. This downward spiral has been shown to lead to failure of renal arterial autoregulation, vasoconstriction as well as decrease in single nephron glomerular filtration rate [10, 11]. 

### Management 

The identification, monitoring and intervention of at-risk patients for TLS follows follow a schematic from the guidelines. 

A 2008 international expert panel on TLS recommended that both children and adults at any risk for TLS initially receive 2 – 3 L/m^2^ per day of IV fluid. There are no specific guidelines for the type of fluid or duration, which should depend on clinical discretion [13]. For adult and pediatric patients with intermediate-risk TLS which include highly chemotherapy-sensitive solid tumors, allopurinol is recommended. Rasburicase is recommended for the initial management of most adult and pediatric patients with high-risk disease. Patients who develop TLS should be monitored in the intensive care unit, assessed for urgent dialysis, and rasburicase continued at 0.2 mg/kg [13]. Furthermore, indications for renal replacement therapy follow the general guidelines for the treatment of AKI and its complications. As in our case, it is probable that the likelihood of renal complete recovery is good if renal replacement therapy is initiated early. 

When our patient developed TLS, which we defined by the classic laboratory criteria and the clinical criteria, we proceeded to dialyze based on massive hyperphosphatemia (21.4 mg/dL), hypocalcemia (corrected: 6.9 mg/dL) with calcium-phosphate product of 149.8, hyperkalemia (6.2 mM/L), in the overall context of acute renal failure (serum creatinine > 1.5 times the upper limit of normal) and oliguria (< 400 mL/day). Our case highlights clinical decision-making involving the known risks of dialysis-related disequilibrium from intermittent dialysis with the anticipated benefits of effectively controlling severe hyperphosphatemia [8]. We utilized an initial intermittent dialysis to achieve initial control of massively elevated phosphate, followed by continuous hemodialysis to manage the continued production of phosphorus from cell lysis. Our protocol was an initial session of conventional hemodialysis, 4-hour session with a blood flow of 200 and dialysate flow of 400 mL/minute; with electrolyte composition measuring 150 mM of Na/L and calcium of 2.5 mEq/L. This was accompanied by a simultaneous mannitol administration of 12.5 gat 2 and 4 hours after the start of hemodialysis. The resulting stability of serum osmolality was demonstrated with a measured osmolality reading of 322 and 319 mOsm/kg before and after, respectively. Serum phosphate improved to 8.7 mg/dL. An alternative pathway of establishing osmotic stability in the presence of high risk for brain edema or dialysis disequilibrium on upfront hemofiltration or hemodialysis involves limited dose 3% saline infusion (2 – 3% of global effluent rate) [8, 14, 15]. In our case, high-volume slow continuous hemofiltration was commenced after hemodialyisis, with an initial hemofiltration rate of 30 mL/kg/hour (2 L/h). The serum phosphate was difficult to control and even rose temporarily within the next 24 hours (13, 11 mg/dL), so the rate of hemofiltration was subsequently further escalated to 3.5 L/h. Continuous arteriovenous hemodialysis with a high flow rate of up to 4 L/h was needed in previous reports with good results and efficacy in the treatment of TLS [16]. In this case, we are also utilizing continuous veno-venous hemofiltration to achieve satisfactory results. No rebound increases in phosphorus or potassium were noticed after the cessation of therapy. The patient showed excellent tolerance for these therapies and eventually recovered renal function as demonstrated during follow-up visits. 

## Discussion 

Hemodialysis has been recommended for life-threatening hyperphosphatemia and other electrolyte disturbances. Conventional hemodialysis removes excessive phosphorus by the process of diffusion. A previous study had shown that after a 4-hour hemodialysis in adults, only ~ 511 ± 222 mg of phosphorus can be removed, and the rate of this removal is directly proportional to the serum concentration, which becomes less as dialysis progresses. Due to the kinetics of phosphorus during dialysis, most of the removal during a typical intermittent hemodialysis will occur during the first half of treatment [17, 18]. Moreover, after a completed hemodialysis session, there is a rebound increase in serum phosphorus and a continued release of phosphorus from dying cells, hence the need for continuous veno-venous hemofiltration. The removal of phosphorus using this modality is dependent on the serum level and the hemofiltration rate, which can be adjusted further as needed by serial measurements of serum phosphorus. We advocate utilizing initial conventional hemodialysis to rapidly decrease the serum phosphate level and the efficacy of continuous hemofiltration for the continued maintenance of controlled phosphate levels during TLS. 

Uric acid nephropathy is considered to be a major cause of AKI in the setting of TLS. As it has a pKa of 5.6, it can precipitate in the renal tubules under generation of acidic urine. The setting of TLS renal arterial vasoconstriction, renal medullary hemoconcentration and decreased tubular flow rates are believed to be additional pathophysiology by means of which elevated uric acid may lead to decreased estimated glomerular filtration rate and promote precipitation [19]. This was commonly caused by forced alkalization of urine, which is no longer recommended. Secondarily, alkalization would further decrease the amount of ionized calcium in the circulation via the increased binding of albumin to calcium, which can also precipitate tetany or seizures and lead to recalcitrant hyperphosphatemia [20]. Xanthinuria has also been identified as a potential cause of AKI, due to the widespread use of hypouricosuric agents, such as allopurinol, that block the metabolism of hypoxanthine and xanthine leading to an increase in xanthine, which can also precipitate if reaching excessive concentration urine, though less than uric acid [21]. Thus, xanthine nephropathy is a non-intended consequence of severe TLS being treated with allopurinol. If this complication is suspected, the medication dosage should be decreased or discontinued in patients who meet the criteria for use but subsequently develop AKI [22]. In contrast to allopurinol, recombinant urate oxidase enzyme rasburicase (trade name: Elitek; Sanofi-Aventis, Sanofi-Aventis, Bridgewater, NJ, U.S. LLC.), which converts uric acid to allantoin, produces a soluble and inactive metabolite of uric acid and is recommended for use in patients with a high risk of TLS. These patients are considered to have > 5% likelihood of TLS, and often present with renal failure [23]. Patients with a high risk of TLS should have treatment with 3 L/m^2^ of IV hydration per day, avoid urinary alkalization, and be given 1 dose of rasburicase (0.1 – 0.2 mg/kg). This treatment is not to be repeated unless clinically necessary. However, in patients with a history of glucose-6-phosphate dehydrogenase deficiency, rasburicase is advisedly contraindicated [24]. Previous peer-reviewed case reports have presented the treatment with continuous hemodiafiltration or continuous arteriovenous hemodialysis as a more potent form of treatment as compared with conventional hemodialysis in controlling and managing acute renal failure and TLS [25, 26]. 

We conclude that continuous veno-venous hemofiltration with high therapy fluid rates up to 3.5 – 4 L/h is a definitive therapy for acute renal failure and hyperphosphatemia in TLS patients who meet the standard criteria for renal replacement therapy. Burkitt’s lymphoma and Burkitt’s leukemia appear to be the commonest causes of TLS with electrolyte disturbances in oncological patients receiving chemotherapy [27]. Although this is a single case, the systematic application of the renal replacement modalities described above early in the course of the disease likely contributed to the achievement of complete recovery in our index patient. 

## Acknowledgment 

Parts of this project have been presented in an online format at ASN Kidney Week 2018 Annual Meeting, October 25 – 28, 2018, San Diego, CA, J Am Soc Nephrol 2018; 29: 1208A. The first draft of this paper was written while Dr. Amaechi still in Nephrology Fellowship at the Medical University of South Carolina (Class of 2018). Dr. Fülöp is current employee of the United States Veterans Health Administration. However, the views and opinions expressed herewith do not reflect the official views or opinion or were endorsed by the FMC Hungary or the United States Veteran Health Administrations. 

We sincerely appreciated the assistance of Mr. Attila Lénárt-Muszka during editing and grammar review. 

## Funding 

None received. 

## Conflict of interest 

No conflict of interest. 

**Table 1. Table1:** Adaptation of Cairo and Bishop criteria for tumor lysis syndrome.

Abnormality	Laboratory criteria	Clinical criteria
Hyperphosphatemia	Phosphorus > 4.5 mg/dL or > 6.5 mg/dL in children	Respiratory failure, hypotension, neuromuscular irritability
Hyperuricemia	Uric acid > 8.0 mg/dL or above upper limit of normal range for age in children	(May contribute to renal failure*)
Hyperkalemia	Potassium > 6.0 mM/L	Cardiac dysrhythmia or sudden death
Hypocalcemia	Corrected calcium < 7.0 mg/dL or ionized calcium < 1.12 mM/L	Cardiac dysrhythmia or sudden death
Acute kidney injury		Increase in serum creatinine by 0.3 mg/dL or > 1.5 times the upper limit of normal or oliguria defined as a urine output < 0.5 mL/kg/hour for 6 hours

*Mechanisms involved in contributing to acute kidney injury and renal failure may include crystalluria and uric acid nephropathy.

**Table 2. Table2:** Examples of tumor lysis syndrome prophylaxis and treatment recommendations based on risk assessment.

Low risk disease	Intermediate risk disease	High risk disease
Indolent non-Hodgkin’s lymphoma, multiple myeloma, chronic myeloid leukemia, acute myeloid leukemia and WBC < 25 × 109/L and LDH < 2 × ULN.	Rare, highly chemotherapy-sensitive solid tumors including neuroblastoma, germ cell tumor, small-cell lung cancer with bulky or advanced stage disease.	Adult T cell leukemia/lymphoma, Burkitt’s leukemia, diffuse large B-cell, transformed, and mantle cell lymphomas with bulky disease and LDH ≥ 2 × ULN, or Burkitt’s lymphoma stage III/IV and/or LDH ≥ 2 × ULN.
Prophylaxis and treatment		
Monitoring Hydration ± Allopurinol	Monitoring Hydration Allopurinol	Monitoring Hydration Rasburicase

*ULN = upper limit of normal; LDH = lactate dehydrogenase; WBC = white blood cells.
